# Surrogate models provide new insights on metrics based on blood flow for the assessment of left ventricular function

**DOI:** 10.1038/s41598-022-12560-3

**Published:** 2022-05-24

**Authors:** Dario Collia, Giulia Libero, Gianni Pedrizzetti, Valentina Ciriello

**Affiliations:** 1 Department of Civil, Chemical, Environmental and Materials Engineering, University of Bologna, 40136 Bologna, Italy; 2 Department of Engineering and Architecture, University of Trieste, 34127 Trieste, Italy

**Keywords:** Cardiology, Engineering

## Abstract

Recent developments on the grading of cardiac pathologies suggest flow-related metrics for a deeper evaluation of cardiac function. Blood flow evaluation employs space-time resolved cardiovascular imaging tools, possibly integrated with direct numerical simulation (DNS) of intraventricular fluid dynamics in individual patients. If a patient-specific analysis is a promising method to reproduce flow details or to assist virtual therapeutic solutions, it becomes impracticable in nearly-real-time during a routine clinical activity. At the same time, the need to determine the existence of relationships between advanced flow-related quantities of interest (QoIs) and the diagnostic metrics used in the standard clinical practice requires the adoption of techniques able to generalize evidences emerging from a finite number of single cases. In this study, we focus on the left ventricular function and use a class of reduced-order models, relying on the Polynomial Chaos Expansion (PCE) technique to learn the dynamics of selected QoIs based on a set of synthetic cases analyzed with a high-fidelity model (DNS). The selected QoIs describe the left ventricle blood transit and the kinetic energy and vorticity at the peak of diastolic filling. The PCE-based surrogate models provide straightforward approximations of these QoIs in the space of widely used diagnostic metrics embedding relevant information on left ventricle geometry and function. These surrogates are directly employable in the clinical analysis as we demonstrate by assessing their robustness against independent patient-specific cases ranging from healthy to diseased conditions. The surrogate models are used to perform global sensitivity analysis at a negligible computational cost and provide insights on the impact of each diagnostic metric on the QoIs. Results also suggest how common flow transit parameters are principally dictated by ejection fraction.

## Introduction

The study of fluid dynamics inside the left ventricle (LV) of the human heart is of considerable interest for the identification of long and short-term pathologies^1,2^. Several studies in the literature have highlighted how flow-mediated metrics participate to the progression or regression of cardiac pathologies^3,4^, making intracardiac fluid dynamics an increasingly integral part of clinical evaluations. Cardiovascular imaging allows, to some extent, to measure cardiac fluid dynamics in vivo with results that are accumulating^5^. To date, various diagnostic techniques are used for the identification of flow-related LV functional properties through the use of tools such as 2D-transthoracic echocardiography, 4D-transesophageal echocardiography, and cardiac magnetic resonance (CMR), even though they present several limitations and are not routinely employed in clinical applications. At the same time, the direct numerical simulation (DNS) of the equation governing blood flow, carefully integrated with the boundary information obtained from routine diagnostic imaging, represents an alternate approach that is becoming popular in clinical research involving intraventricular fluid dynamics^6–8^; this approach is demonstrating usefulness for analyzing patient-specific cardiac flow conditions^2,9^. DNS also represents a viable tool to integrate existing imaging technology and extend it with the capability of reproducing flow details in virtual conditions corresponding to hypothetical therapeutic solutions^10^.

The standard clinical assessment of cardiac pathology is based on a series of quantitative metrics that are directly or indirectly related to LV flow. First, the ejection fraction (EF), i.e. the reduction of LV volume normalized with the initial value, represents the main measure of myocardial contraction and it is critical to describe the cardiac systolic LV function^11^. Another clinical metric commonly employed to describe diastolic function is the ratio E/A^12^; it is defined as the ratio between the early (E-wave) and atrial (A-wave) peaks of the mitral inflow velocity, that is measured from pulsed-wave Doppler. This metric is used—in conjunction with others- for the classification of diastolic dysfunction as it reveals to which degree the LV fills passively during the early phase of diastole and how much LV filling must rely on the active support of atrial contraction to be completed^13,14^. As for LV geometry, the ventricular length and diameter measured at the end of diastole (End-Diastolic-Length, EDL, and End-Diastolic-Diameter, EDD) are also important determinants of the ventricular function^15^. All such metrics affect intracardiac blood flow although they do not represent direct measurements of fluid dynamics phenomena. More sophisticated tools and methods, such as 4D flow magnetic resonance imaging (MRI) and DNS, allow identifying flow-specific quantities that provide a more profound description of intraventricular fluid dynamics. For example, the kinetic energy (KE) is a quantitative measure of LV blood flow vitality particularly during diastolic filling^16,17^. The vorticity field, and its average value $\overline{\omega }$, plays a central role in the description of the flow patterns throughout the cardiac cycle^18–20^; indeed, vortices represent the underlying structure of intraventricular flow and they are crucial for stability and for the dynamic balance between rotating blood and myocardial tissue^1,21^. The description of blood transit through the LV is also fundamental in cardiovascular physiology^22^ to identify properties of blood transport and mixing inside the LV in order to assess the residence time and the rapidity of blood wash-out; phenomena that can be related to the risk of thrombus formation^10,23^.

The objective of this study is to explore the existence of relationships and dependencies between advanced flow-related quantities and the metrics used in the standard clinical assessment of cardiac function. This can lead on the one side to recognize the role (if any) of clinical metrics for describing blood flow properties, and on the other to identify the incremental value (if any) provided by more advanced flow analysis.

To answer these questions we apply surrogate models based on the Polynomial Chaos Expansion (PCE) technique^24,25^. These surrogates are a class of reduced-order models (ROMs) that allow decreasing the complexity of a given high-fidelity model (HFM) by learning the dynamics of a quantity of interest (QoI) directly from the HFM’s response. As a consequence, the surrogate is able to capture the key features of the underlying dynamics for the specific QoI; at the same time, the high computational cost of physics-based simulations performed with the HFM is drastically reduced to a limited number of runs necessary to calibrate the surrogate model. This allows to perform in-depth analyses (otherwise unfeasible on the HFM) such as global sensitivity analysis (GSA) and Monte Carlo simulations (MCS) at a negligible computational cost (e.g.,^26,27^). The use of PCE-based surrogates in cardiovascular research is becoming popular for uncertainty quantification purposes to provide relevant information to assist the clinical practice. As an example, in the context of cardiovascular numerical simulations, the stochastic collocation method is used to propagate uncertainty from clinical data to the predictions of hemodynamic quantities and surgical outcomes in^28,29^; while in^30^, uncertainty quantification based on the PCE is applied to 3D patient-specific cardiovascular models.

This study combines numerical simulations of blood flow in the LV with PCE surrogates to provide new insights on the fundamental understanding of the LV function. In the proposed framework of analysis, the HFM is given by a DNS method of intraventricular fluid dynamics that allows to simulate and analyze cardiac flow in individual patients. Numerical simulations are based on the immersed boundary method (IBM)^31,32^ where flow equations are solved in a regular Cartesian grid with a distribution of fictitious forces concentrated at the immersed solid boundaries (whose intensity is matched to ensure fulfillment of the boundary conditions); this computational approach is often employed to analyze LV fluid dynamics^6,33^. In the same framework, the dynamics of the mitral valve (MV) are reproduced by a kinematic model that was introduced and validated in previous studies^7,8^.

Surrogate models are used to approximate two QoIs describing LV blood transit as (i) the direct volume, hereinafter $V_d$, which is the volume of blood that enters the LV and is ejected during the same heartbeat, and (ii) the residual volume, hereinafter $V_r$, which is the amount of blood that is present in the LV at the beginning of diastole and is not ejected during systole; both volumes are expressed in dimensionless form as a percentage of the End Diastolic Volume (EDV)^22^. Surrogates are also computed for the following two dynamic QoIs: (iii) the ventricular kinetic energy, and (iv) the vorticity, both measured at the peak of early diastolic filling, hereinafter $KE_{Epeak}$ and $\overline{\omega }_{Epeak}$. Both quantities are made dimensionless using the mitral velocity (${E_{peak}}$), LV volume and, for the latter only, the MV diameter. Note that instantaneous values at the peak of early diastole may not provide an exhaustive description of the complex intraventricular flow pattern. Nevertheless, the values at the peak of the E-wave are considered a good reference as they are commonly assumed to well describe the diastolic function^20,21,34^. The core of the vortex ring develops from the beginning of the early filling and reaches its full development when this phase approaches its peak^21^. Recent studies have shown that at the E-wave peak the vortex is well-formed and differentiates healthy from pathological conditions^8,10,20^.

The response surfaces of the QoIs are approximated in the space of four parameters that we select among the diagnostic clinical metrics describing the overall LV health. These parameters embed relevant information on LV geometry and function and help discriminating healthy and diseased patients in numerous clinical conditions. The selected parameters are the ejection fraction, *EF* = $(EDV-ESV)/EDV$; the ratio *E*/*A*; the LV shape ratio $LV_r$ = EDL/*EDD* and the ratio $MVLV_r$ = $MV_a/LV_a$ between the diameter of the MV annulus ($MV_a$) and that of the entire LV valvular plane ($LV_a$), which is useful for the correct identification of the MV orifice size^35^ and the transformation between LV size and blood velocity under different ventricular conditions^20^.

Calibration and validation of surrogate models is preformed on different datasets generated by running the HFM multiple times for a parameterized geometry that can be altered to arbitrary values of the governing clinical parameters. GSA is then performed for each QoI to assess the relative impact of the variability of the governing parameters. In addition, we assess the robustness of the predictions provided by the surrogates in comparison with results obtained by DNS (with the HFM) for several patient-specific cases under both healthy and diseased conditions. Finally, the application of surrogate models in the present study allows us to uncover how common global flow transit quantities are related to standard clinical parameters.

## Results

### Computation of the surrogate models

We consider the set of QoIs $\left\{ V_d, V_r, KE_{Epeak}, \overline{\omega }_{Epeak} \right\}$ provided by the HFM, i.e. by DNS solving the Navier-Stokes equation to mimic intraventricular fluid dynamics in individual patients^8^. Based on preliminary analysis against patient-specific cases, we identify four governing parameters, namely *EF*, *E*/*A*, $LV_r$, $MVLV_r$, able to embed key information on LV geometry and function. We provide a probabilistic description for the governing parameters by using uniform distributions, with ranges of variation covering both healthy and diseased conditions^15,35–41^ (see Table 1). A parameterized geometry, based on the combination of two patient-specific cases and varying only as a function of the governing parameters, is used for the purpose of generating the surrogate models.

**Table 1:** Probabilistic distributions associated with the selected governing parameters.

Parameter	Distribution
*EF*	$\mathrm {U} [0.3,0.7]$
*E*/*A*	$\mathrm {U} [0.5,2.0]$
$LV_r$	$\mathrm {U} [1.0,2.0]$
$MVLV_r$	$\mathrm {U} [0.4,0.7]$

The PCE approximation of each QoI is realized in the space generated by $M=4$ independent random variables, $\xi _i$, uniformly distributed in $[-1;1]$; $\xi _i$, with $i=1,\ldots ,4$, are associated with the governing parameters through an isoprobabilistic transform^42^. As a consequence, the generic QoI ${\hat{y}}_k \in \left\{ V_d, V_r, KE_{Epeak}, \overline{\omega }_{Epeak} \right\}$ is approximated as follows:

1 $$\begin{aligned} {\hat{y}}_k = \sum _{j=0}^{P-1} s_j \Psi _j (\xi _1,\xi _2,\xi _3,\xi _4), \end{aligned}$$

where $\Psi _j$ are second-order ($q=2$) multivariate Legendre polynomials, $P=( M+q )!/(M!q!)=15$, and $s_j$ are the deterministic coefficients computed by running the HFM for $N=P$ sampling points identified by the probabilistic collocation method (PCM)^24,25,43^. Coefficients $s_j$ for each QoI are collected in Table 2.

**Table 2:** PCE coefficients for each QoI.

QoI	$V_d$	$V_r$	$KE_{Epeak}$	$\overline{\omega }_{Epeak}$
$s_0$	3.23E-01	3.23E-01	4.50E-01	6.02E+00
$s_1$	1.62E-01	− 2.38E-01	2.07E-01	2.33E+00
$s_2$	5.16E-03	5.16E-03	9.62E-02	1.28E+00
$s_3$	− 1.87E-02	− 1.94E-02	− 5.81E-03	− 6.24E-01
$s_4$	1.29E-02	1.29E-02	4.78E-02	6.69E-01
$s_5$	1.61E-02	1.56E-02	6.11E-02	6.08E-01
$s_6$	3.33E-03	3.33E-03	− 2.83E-02	− 1.87E-01
$s_7$	3.33E-03	3.33E-03	− 5.33E-02	− 6.58E-01
$s_8$	1.00E-02	1.00E-02	1.17E-02	− 3.15E-01
$s_9$	− 7.78E-03	− 6.67E-03	− 2.61E-02	− 9.67E-02
$s_{10}$	5.00E-03	3.33E-03	3.33E-03	1.17E-01
$s_{11}$	2.00E-02	2.00E-02	8.50E-02	− 1.38E-01
$s_{12}$	− 1.67E-03	− 1.11E-03	− 1.67E-03	1.77E-01
$s_{13}$	6.67E-03	5.00E-03	4.50E-02	9.33E-02
$s_{14}$	− 1.11E-03	− 1.11E-03	− 1.22E-02	7.61E-02

**Figure 1:**
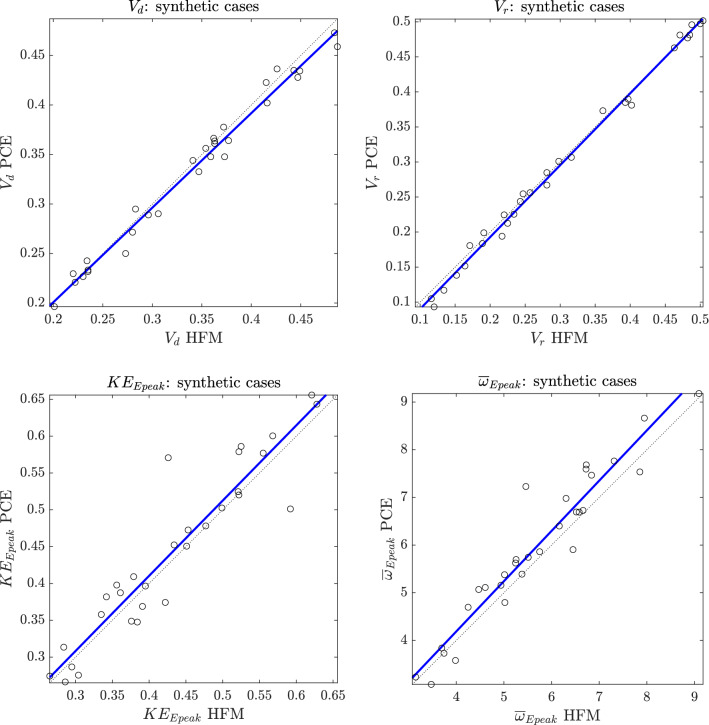
Comparison between HFM and PCE predictions against synthetic cases. Solid blue lines represent the linear regressions, while dashed lines are the bisectors. Characteristics of regression lines: $$V_d \mathrm {PCE} = 0.95 \cdot V_d \mathrm {HFM} + 0.0099$$ $$(\mathrm {R^2}=0.98, p-$$value$$= 9.74$$E$$-27)$$; $$V_r \mathrm {PCE} = 1.00 \cdot V_r \mathrm {HFM} -0.012$$ $$(\mathrm {R^2}=0.99, p-$$value$$= 3.29$$E$$-33)$$; $$KE_{Epeak} \mathrm {PCE} = 1.00 \cdot KE_{Epeak} \mathrm {HFM} + 0.0018$$ $$(\mathrm {R^2}=0.87, p-$$value$$= 4.68$$E$$-14)$$; $$\overline{\omega }_{Epeak} \mathrm {PCE} = 1.10 \cdot \overline{\omega }_{Epeak} \mathrm {HFM} -0.029$$ $$(\mathrm {R^2}=0.92, p-$$value$$= 1.50$$E$$-16)$$.

The accuracy of the surrogate approximations, with respect to the HFM, has been then verified against 30 newly simulated cases (synthetic cases of LV based on the parameterized geometry with random combination of parameters). Comparative results are shown in Fig. 1. Values of $R^2$, as well as the equations of the regression lines for each QoI, denote the high reliability of the proposed method.

### Sensitivity analysis

Once the PCE approximations of the QoIs are available, GSA on the surrogate models is performed at a negligible computational cost^27^.

**Figure 2:**
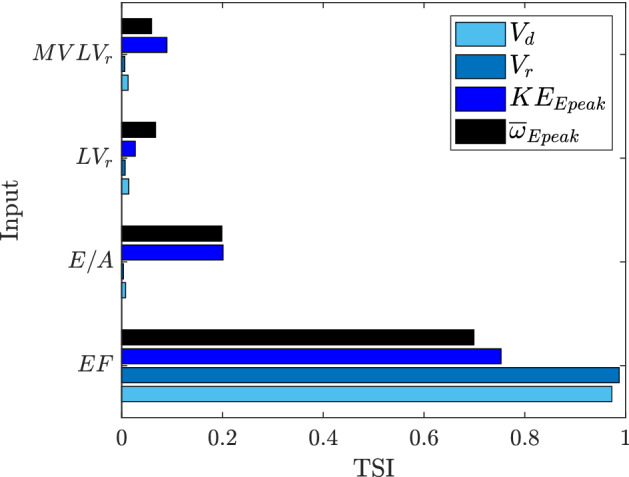
Total sensitivity indices (TSI) of Sobol.

The sensitivity indices of Sobol^44^ are computed as analytical post-processing of the PCE coefficients^42^, for the inputs *EF*, *E*/*A*, $LV_r$, and $MVLV_r$, with respect to each QoI, i.e. $V_d$, $V_r$, $KE_{Epeak}$ and $\overline{\omega }_{Epeak}$. In particular, in Fig. 2 the total sensitivity indices (TSI) are shown; the sum of the TSI for each QoI is slightly higher than unity, thus indicating negligible second-order effects^44^. The parameter which explains more the variability of all the selected QoIs is *EF*; specifically, the variance of $V_d$ and $V_r$, is almost totally attributable to the variability of *EF*. As for $KE_{Epeak}$ and $\overline{\omega }_{Epeak}$, if *EF* reflects about the $75\%$ and $70\%$ of their variance respectively, *E*/*A* plays a secondary role (about $20\%$ of the variance of both the QoIs), while the influence of $LV_r$ and $MVLV_r$ is lower ($\le 10\%$).

### Surrogate predictions of patient-specific cases

The accuracy provided by the surrogate models is further explored by using them to reproduce left ventricular flow parameters in a series of real, patient-specific, cases. These geometries are recorded through 4D transesophageal echocardiography where the geometric data is extracted with the use of dedicated software (4D LV-Analysis, 4D MV-Assessment, TomTec Imaging Systems GmbH, Unterschleissheim, Germany).

**Figure 3:**
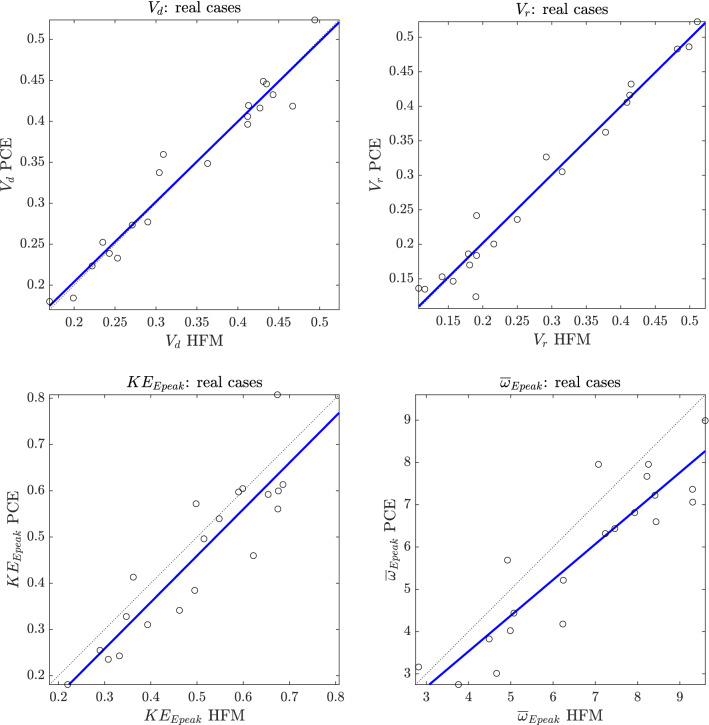
Comparison between HFM and PCE predictions against real cases. Solid blue lines represent the linear regressions, while dashed lines are the bisectors. Characteristics of regression lines: $$V_d \mathrm {PCE} = 0.98 \cdot V_d \mathrm {HFM} + 0.0084$$ $$(\mathrm {R^2}=0.95, p-$$value$$= 2.41$$E$$-13)$$; $$V_r \mathrm {PCE} = 0.99 \cdot V_r \mathrm {HFM} + 0.0041$$ $$(\mathrm {R^2}=0.97, p-$$value$$= 6.41$$E$$-15)$$; $$KE_{Epeak} \mathrm {PCE} = 1.00 \cdot KE_{Epeak} \mathrm {HFM} -0.043$$ $$(\mathrm {R^2}=0.82, p-$$value$$= 4.98$$E$$-08)$$; $$\overline{\omega }_{Epeak} \mathrm {PCE} = 0.85 \cdot \overline{\omega }_{Epeak} \mathrm {HFM} + 0.15$$ $$(\mathrm {R^2}=0.82, p-$$value$$= 4.23$$E$$-08)$$.

Figure 3 compares the predictions of each QoI obtained with the HFM and the surrogate for these 20 cases, and provides the characteristics of the correspondent regression lines (equations and $R^2$). Despite being calibrated against a parameterized geometry, the surrogates demonstrate to provide robust predictions also when compared to independent patient-specific simulations run with the HFM. In particular, it is noted that slope values of the regression lines are almost equal to 1 for $V_d$, $V_r$ and $KE_{Epeak}$ and that the $R^2$ value is above 0.8 for all the QoIs. Even $\overline{\omega }_{Epeak}$, whose dynamic is more complicated to capture, is quite accurately represented by the correspondent surrogate model.

## Discussion

The description of the cardiac mechanical function is commonly performed by measuring a series of geometrical and physical parameters (e.g., volume reduction or ventricular pressure) that represent physical phenomena describing the cardiac activity. Recent years have witnessed rapid advancements in imaging technology and methods of image analysis that provide deeper informative content regarding cardiac function. By first principles, heart function is about creating and sustaining blood motion; therefore, placing the focus on measures associated with blood flow promises further progress along this line^1,45^.

Imaging technology dedicated to blood flow (e.g. 4D Flow MRI) presents significant costs and complexity making it not appropriate to evaluations performed during the clinical routine. On the other hand, clinical assessments of blood flow can only be based on a few global, integral parameters and do not require explicit knowledge of the time- and space-resolved three-dimensional velocity vector field. This study presents, for the first time, the application of surrogate models to LV blood flow, calibrated on a series of statistically selected flow fields, to reproduce global parameters that are suggested to be of clinical interest.

The proposed surrogate model is characterized by simplicity and reproducibility, making it appropriate for straightforward application in clinical routine; it demonstrates a significant accuracy when tested on 20 patient-specific (real) cases, by providing predictions very close to the results obtained with the DNS. This accuracy is excellent when the surrogate model is employed to evaluate the flow transit across the ventricle in terms of direct and residual flows.

The evaluation of transit volumes receive considerable attention in the clinical literature for their potential clinical relevance as a risk factor for thrombus formation and decision for anticoagulation therapy. In literature, these parameters are mainly evaluated by 4D Flow MRI and report that LVs with a reduced *EF* typically present a worsening of flow transit characterized by reduction of the direct flow accompanied by an increase of the residual volume. The excellent reliability of the surrogate model (Fig. 3), jointly with the dominant role played by *EF* in the model itself (Fig. 2), suggest that *EF* may represent a key factor in determining the values of the transit flow parameters.

**Figure 4:**
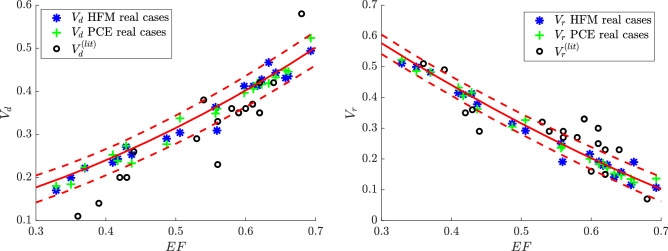
$V_d$ and $V_r$ against *EF* for the 20 patient-specific (real) cases analysed in this study and based on the predictions provided by the HFM and the PCE-based surrogate models. Other cases derived from literature, $V_d^{(lit)}$ and $V_r^{(lit)}$, are also represented. The red continuous line represents the mean of the QoI (either $V_d$ or $V_r$) against *EF* obtained with the surrogate model, given the variability of the remaining parameters (red dashed lines represent the 1% and 99% quantiles).

It is possible to observe that the surrogate models suggest a sensibly linear relationship for both $V_d$ and $V_r$ as functions of *EF* (see Table 2). These relationships are shown in Fig. 4 where the average (solid) curve with 1% and 99% quantiles (dashed) is reported for the two QoIs ($V_d$ and $V_r$), accounting for the variability of the remaining parameters (i.e. *E*/*A*, $LV_r$, and $MVLV_r$). The same Figure reports the values relative to the 20 patient-specific (real) cases as obtained by the HFM and the PCE-based surrogate models, which underline the robustness of this method. The pairs of $(EF,V_d^{(lit)})$ and $(EF,V_r^{(lit)})$ as extracted directly from the literature^23,34,46–54^ are also represented. These literature data match with the results provided by the proposed method up to a certain degree, although a noticeable discrepancy is detected. This difference can be preliminarily imputable to the presence of regurgitation, which is not present in the HFM cases employed for the calibration (as such it can’t be captured by the surrogate models). The difference should be partly imputable to the simplifying assumptions that are present in the numerical model; it can also be attributed -in some cases- to the limited accuracy of the calculation during imaging post-processing that are non-trivial (possibly performed with commercial software) and that sometimes gives rise to non-physical differences between LV inflow and outflow measurements. A more consistent comparison of literature data with our findings can be performed by correcting the literature data taking into account the balance of mass between inflow and outflow. This balance, even in presence of regurgitation, corresponds to an exact relationship between the direct and residual volumes

2 $$\begin{aligned} V_d - V_r = 2 EF-1. \end{aligned}$$

**Figure 5:**
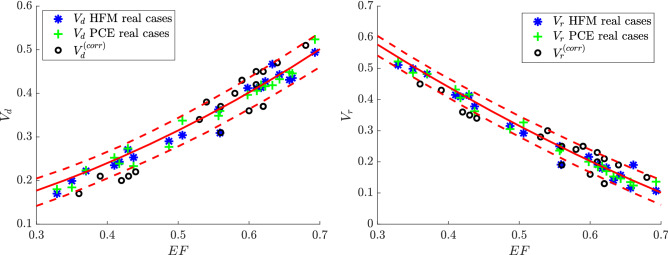
$V_d$ and $V_r$ against *EF* for the 20 patient-specific (real) cases analysed in this study and based on the predictions provided by the HFM and the PCE-based surrogate models. Other cases derived from literature, $V_d^{(corr)}$ and $V_r^{(corr)}$, are also represented in their modified version after correction for the regurgitating volume. The red continuous line represents the mean of the QoI (either $V_d$ or $V_r$) against *EF* obtained with the surrogate model, given the variability of the remaining parameters (red dashed lines represent the 1% and 99% quantiles).

A simple (blind) manner to ensure fulfillment of this constraint in the empirical literature data is estimating theoretical values based on (2), i.e. $V_d^{(th)}=V_r^{(lit)}+2 EF-1$ and $V_r^{(th)}=V_d^{(lit)}-2 EF+1$. Then, the error is equally distributed on both to obtain corrected values: $V_d^{(corr)}=(V_d^{(lit)}+V_d^{(th)})/2$ and $V_r^{(corr)}=(V_r^{(lit)}+V_r^{(th)})/2$. Note that the corrected values satisfy the balance (2), without making any specific assumption on the possible origin of the discrepancy; therefore the correction can be acceptable until the error of the balance given by (2) for the original data is not excessive in relative terms. Figure 5 presents the same results of Fig. 4 with the corrected values of literature data $V_d^{(corr)}$ and $V_r^{(corr)}$. The agreement is noticeably improved and confirms the existence of a dominant influence of the value of *EF* on the global flow transit parameters in a wide range of LV conditions.

The finding that *EF* is the main determinant for overall quality of blood transit across the LV is in agreement with the common clinical knowledge, in absence of atrial fibrillation, for the decision of anticoagulant therapy^55^. It must be remarked, however, that this does not invalidate the need for a blood flow analysis in the cardiac chambers. It rather stresses that simple global properties, like $V_d$ and $V_r$, may not require a thoughtful evaluation of blood flow details; nevertheless, individual thrombus formations are often associated with regional ischemia^56,57^. In such cases, physics-based analysis of space-time-extended transit properties of intraventricular blood flow may be required to assess risk factors at the individual level and improve screening and prevention.

This study suggests how some common properties associated with blood flow can be estimated with surrogate models based on standard clinical parameters. Such surrogate models require a preparatory effort for the initial calibration (DNS or 4D Flow MRI), then they present a notable simplicity and can be employed in real-time during the clinical analysis.

Merging surrogate models and DNS represents a promising winning approach to describe the most important metrics in the cardiology field. In this exploratory study, we analyze the most common cardiological parameters such as *EF*, *E*/*A*, $LV_r$, $MVLV_r$, taking into account that the valvular dimension is also expected to have a direct influence on intraventricular flow. The selected parameters are commonly used in the context of diagnostic examination and clinical evaluation^11–15^, especially in presence of diastolic dysfunction where the flow can play a role; in addition, they can be considered, in the first instance, as independent. Our findings are the basis for more in-depth assessments involving further parameters to account for the regional function, synchrony, and deformation properties. Many of such parameters, however, are closely correlated to the value of EF^58^ that is preferred here as a starting point. As such, in future work, we aim to extend the analysis to other clinical parameters, possibly even in substitution of EF, by separating the properties concurring with the definition of its value. In a broader perspective, this work aims to suggest a method for a combined analysis of multiple clinical evaluations in a possible direct and non-invasive way.

## Materials and methods

### High-fidelity model

#### Geometric description

**Figure 6:**
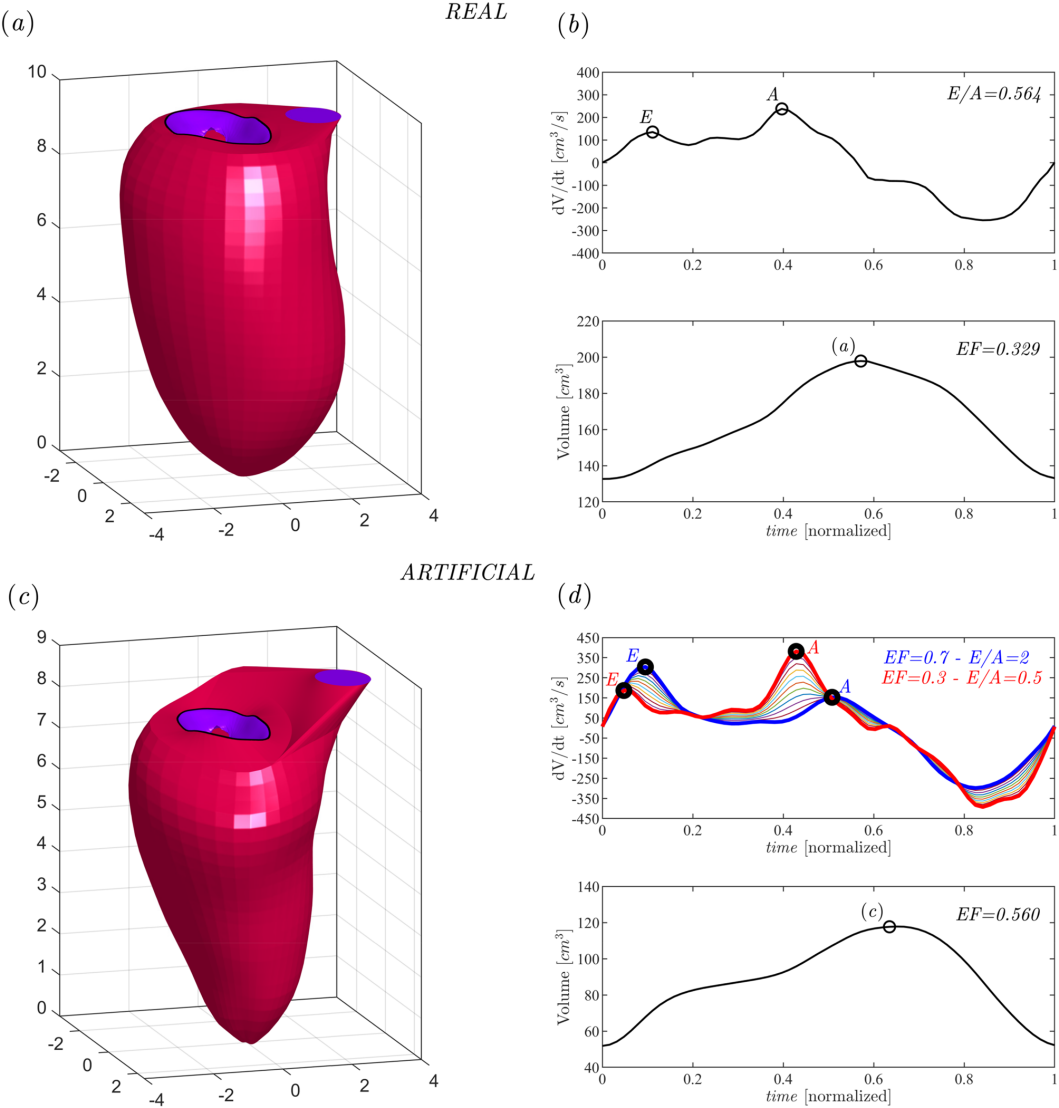
First row: panel (**a**) shows the LV geometry for a patient-specific case; panel (**b**) shows the correspondent volume curve and *dV*/*dt* with values of *EF* and *E*/*A* displayed. Second row: panel (**c**) depicts a synthetic LV obtained with the parameterized geometry; panel (**d**) depicts the correspondent volume curve and *dV*/*dt* where the general approach for the construction of the synthetic cases for given values of the governing parameters is exemplified.

The time-varying geometry of LV is extracted from 3D echocardiography; the moving borders are obtained by a semi-automatic procedure within a dedicated software (4DLV analysis, Tomtec Imaging Systems GmbH, Unterschleissheim, Germany). Subsequently, LV geometry during all phases of the heartbeat is described by the position vector $\mathbf{X} (\vartheta ,s,t)$ of its endocardial surface, where the structured parametric coordinates $(\vartheta ,s,t)$ run along the circumference and from base to apex, respectively, and *t* is time^59^. The position vector marks the points of the LV material, and their velocity is obtained from temporal differentiation. Instead, MVs geometries are extracted from the images with the use of dedicated software (4DMV-Assessment, TomTec Imaging Systems GmbH, Unterschleissheim, Germany), limitedly to the completely open (at the peak of diastole) and completely closed (at the peak of systole) positions, these open positions are described parametrically by the degree of opening of each of the two leaflets, $\varphi _1$ and $\varphi _2$, respectively for the anterior and posterior leaflets, ranging from zero (closed leaflet) to $\frac{\pi }{2}$ (fully open). The extracted MV geometries are rearranged for convenience in terms of another pair of parametric coordinates, $\mathbf{X} _{v}(\vartheta ,s,\varphi _1,\varphi _2)$, where *s* range runs along the circumference and extends from the ring at the trailing edge^8^.The present numerical method has been extensively validated in previous studies from the computational point of view^7,8,20^, and by general comparison with clinical information^2,10,60,61^. Moreover, the global quantities that are used in this work, have been previously methodologically validated and satisfactorily compared with results obtained in clinical cases with advanced imaging techniques^2,4,8,10,16,20,23,34,39,47–49,60–62^. For this study, we use both patient-specific geometries (recorded and extracted as described above) and a parameterized geometry to calibrate the surrogate models. The geometries used for calibration purposes are created from the combination of two patient-specific geometries in healthy and pathological conditions, respectively. These two geometries are extracted and processed as previously described to obtain two reliable physiological and pathological borderline cases, according to the parameters used in the study. The parameterization of the ventricle occurs through the interpolation of the two borderline cases and, according to an independent search system, values of the parameters are automatically detected (one at a time) until the desired final geometry is obtained. As such, each geometry defined through this parametrization method is generated by imposing values of *EF*, *E*/*A*, $LV_r$, $MVLV_r$ , that are set by the user among a considerable series of options (e.g., for *EF* between 0.7 and 0.3). As an example of the geometries used in this study, Fig. 6 panel (a) shows a patient-specific case; this is a pathological case as can be deduced from the correspondent values of *EF* and *E*/*A* in panel (b) where the volume curve and *dV*/*dt* are represented; in panel (c), an example of a synthetic healthy case build with the parameterized geometry is shown, while panel (d) shows the correspondent volume curve and *dV*/*dt*, and exemplifies the parametrization method for the construction of the synthetic cases that we adopted. Specifically, Fig. 6 panel (d) shows *dV*/*dt* for the two borderline cases associated with maximum values of *EF*=0.7 and *E*/*A*=2 (blue curve, healthy case), and minimum values of *EF*=0.3 and *E*/*A*=0.5 (red curve, pathological case); within these two curves, all the synthetic cases generated for given values of the governing parameters are included. Note that the *EDD* and *EDL* values are imposed by modifying the geometry in the *x*, *y*, and *z* axes, while the $MVLV_r$ value is identified by varying the size of the valve annulus as a function of the ventricular one.

The images of the human subjects were recorded at the Cardiovascular Department of the Azienda Sanitaria Universitaria Giuliano Isontina, Trieste. The geometries were provided in anonymous form for the numerical study. All procedures involving human subjects have been performed in accordance with the Declaration of Helsinki, and under the approval of the Ethics Committee of the University of Trieste (protocol no. 0025052).

#### Fluid dynamics

The numerical method is extensively described and validated in a dedicated methodological study^8^, where the valvular dynamics is compared with that obtained by a complete fluid-structure interaction^7^. In this section, we briefly recall the main points of the method used. The intraventricular fluid dynamics is evaluated by numerical solution of the Navier-Stokes and continuity equations

3 $$\begin{aligned}&{{{\frac{\partial {{\varvec{{{v}}}}}}{\partial {t}}}}+{{\varvec{{{v}}}}} \cdot \nabla {{\varvec{{{v}}}}}} = -\nabla p + \nu \nabla ^2 {{\varvec{{{v}}}}}, \end{aligned}$$

4 $$\begin{aligned}&\nabla \cdot {{\varvec{{{v}}}}} = 0; \end{aligned}$$

where ${{\varvec{{{v}}}}}(t,\mathbf{x} )$ is the velocity vector field, $p(t,\mathbf{x} )$ is the kinematic pressure field and $\nu$ is the kinematic viscosity (assumed $0.04 cm^2/s$) of a Newtonian fluid. Solution is achieved by the immersed boundary method in a bi-periodic Cartesian domain as described in previous studies, e.g.^8,31,63^. Time advancement is achieved using a fractional step method as follows. Velocity is preliminarily advanced in time by the Navier-Stokes Eq. (3) using a low-storage, third-order Runge-Kutta explicit scheme. This preliminary velocity, say ${{\hat{{\varvec{v}}}}}$, does not satisfy the incompressibility constraint (4), is corrected by adding a potential field ${\delta {{\varvec{{{v}}}}}=\nabla {q}}$, such that ${{\varvec{{{v}}}}}={{\hat{{\varvec{v}}}}}+{{{{\delta {{\varvec{{{v}}}}}}}}}$ satisfies the continuity and the boundary conditions. The correction potential is found by solution of the Poisson equation

5 $$\begin{aligned} \nabla ^2 q=-\nabla \cdot {{\hat{{\varvec{v}}}}}; \end{aligned}$$

and pressure is updated with *q* accordingly. In the IBM, explicit boundary conditions are only required at the edge of the computational box: they are set periodic in the *x* and *y* directions, while they are zero pressure and normal velocity on the upper and lower ends along *z*, respectively. The 2D periodicity permits a fast solution of the Poisson Eq. (5) through Fourier decomposition as a sequence of tridiagonal systems for each harmonic. Then a no-slip condition is imposed on the immersed boundaries^8,60^. These comprise the ventricle geometry and valves surface. In addition, two cylindrical regions are added extending from a region around the mitral valve (bounded by the LV edge on the mitral side and the curve separating MV and aortic valve) and from around the aortic valve to the upper edge of the computational domain; these additional boundaries represent surrogates of atrium and aorta. They are included for numerical convenience to avoid interference between the outflow and the inflow outside the LV and to avoid nonphysical sharp corners at the edge of the LV basal plane. The overall numerical implementation is extensively validated in previous studies^2,8,10,20,60,61,63^. In this study, it is used a bi-periodic domain with a grid made 128$\times$128$\times$160 points and 8192 time steps. A sensitivity analysis of the Cartesian grid is performed in a series of previous methodological studies based on grid-refinement^8,20^. Additionally, it is also verified that the spatial and temporal resolutions employed here ensure a perfect relationship between the flow rate measured across the LV valves and the volumetric variation *dV*/*dt* in a series of conditions^2^. Once the parametric description of the model $\mathbf{X} _{v}(\vartheta ,s,\varphi _1,\varphi _2)$ is obtained, the dynamic equation of the leaflet opening angle is deduced from the constraint that the motion of the leaflet surface must correspond to the velocity of the fluid in the position of the same surface; a complete description and verification of the computational method, including comparison with a fluid-structure interaction model with a given set of tissue parameters, is reported elsewhere^8^. The leaflet dynamics are obtained by least-squares minimization of the difference, integrated over the valvular surface $A_v$, between the fluid and the valve velocity component normal to the valvular surface. The result is a 2$\times$2 linear system,

6 $$\begin{aligned} \begin{bmatrix} \iint _{A_v} \left( \frac{\partial \mathbf{X} _{v}}{\partial \varphi _1} \cdot {\varvec{{n}}}\right) ^{2} dA &{} \iint _{A_v} \left( \frac{\partial \mathbf{X} _{v}}{\partial \varphi _1} \cdot {\varvec{{n}}}\right) \left( \frac{\partial \mathbf{X} _{v}}{\partial \varphi _2} \cdot {\varvec{{n}}}\right) dA \\ \iint _{A_v} \left( \frac{\partial \mathbf{X} _{v}}{\partial \varphi _1} \cdot {\varvec{{n}}}\right) \left( \frac{\partial \mathbf{X} _{v}}{\partial \varphi _2} \cdot {\varvec{{n}}}\right) dA &{} \left( \frac{\partial \mathbf{X} _{v}}{\partial \varphi _2} \cdot {\varvec{{n}}}\right) ^{2} dA \end{bmatrix} \times \begin{bmatrix} \frac{\partial \varphi _1}{\partial t} \\ \frac{\partial \varphi _2}{\partial t} \end{bmatrix} = \begin{bmatrix} \iint _{A_v} (\textit{v} \cdot {{\varvec{n}}}) \left( \frac{\partial \mathbf{X} _{v}}{\partial \varphi _1} \cdot {{\varvec{n}}}\right) dA \\ \iint _{A_v} (\textit{v} \cdot {{\varvec{n}}}) \left( \frac{\partial \mathbf{X} _{v}}{\partial \varphi _2} \cdot {{\varvec{n}}}\right) dA \end{bmatrix} , \end{aligned}$$

for the two unknowns $\frac{\partial \varphi _1}{\partial t}$ and $\frac{\partial \varphi _2}{\partial t}$, where $\textit{v}$ is the fluid velocity and ${{\varvec{n}}}$ the local normal to valvular surface. The aortic valve, which are downstream of the LV flow fields, are modeled as a simple orifice with a surface that is open or closed. The aorta is considered open when the mitral valve is closed and the normal velocity, averaged over the position of the aortic valve surface, before setting boundary conditions, is directed outward. More details are given in^8^.

#### Vorticity and kinetic energy

The formation of the vortex and its orientation inside the ventricle influence the correct course of the flow throughout the cardiac cycle until its expulsion^18,19,33^. The computation of the average vorticity inside the ventricle is

7 $$\begin{aligned} \overline{\omega } =\frac{1}{V}\int _{V}\vert \varvec{\omega }\vert dV \end{aligned}$$

where *V*(*t*) is the ventricular volume, $\varvec{\omega }(t)=\nabla \times {\varvec{{v}}}$ is the vorticity vector field. The numerical method is based on a staggered grid where, for each cell, the velocity components normal to each face are defined at the center of the face; therefore, the vorticity components are calculated numerically at the midpoints of each edge as the circulation produced by the corresponding velocity components. The KE of the blood is a fundamental component of the work done by the two ventricles, indicated as the movement of the blood within them^16,64^ and is computed as follows

8 $$\begin{aligned} KE(t)=\frac{\rho }{2}\int _{V}v^{2} \, dV, \end{aligned}$$

where $\rho$ is the blood density. We then compute dimensionless QoIs, $KE_{Epeak}$ and $\overline{\omega }_{Epeak}$, as follows: $KE_{Epeak}$ is equal to the $E_{peak}$ of *KE* (Eq. 8), normalized with $\rho$, the square of the transmitral inflow $E_{peak}$ and LV volume; while $\overline{\omega }_{Epeak}$ is equal to the $E_{peak}$ of $\overline{\omega }$ (Eq. 7), normalized with the transmitral inflow $E_{peak}$, the MV diameter and LV volume.

#### Flow transit

The study of the flow transit is an important step for the evaluation of blood transport and mixing inside the LV, this is useful for the identification of the blood residences and wash-out properties; conditions that are related to the risk of the thrombus formation. Following the established literature in cardiology^22,23,45^, this evaluation is obtained through the subdivision of EDV in four sub-volumes

9 $$\begin{aligned} EDV=V_{direct} + V_{delayed} + V_{retained} + V_{residual}, \end{aligned}$$

where $V_{direct}$ (also indicated as $V_d$) is the volume of blood that entered during diastole and transits directly to the aortic outlet during systole, thus residing less than one heartbeat in the LV. $V_{delayed}$ is the quantity of blood already present in the LV at the beginning of diastole and ejected during the following systole. $V_{retained}$ is the amount of blood volume that enters during diastole and is retained in the ventricle to be expelled on the next heartbeat. Finally, $V_{residual}$ (also indicated as $V_r$) is present in the LV before the beginning of diastole and remains in the chamber after the end of systole. These sub-volumes are obtained through blood transport analysis performed by solving the transport-diffusion equation for a passive scalar

10 $$\begin{aligned} {\frac{\partial C}{\partial {t}}+ {{\varvec{v}}}} \cdot \nabla C =v\nabla ^{2}C, \end{aligned}$$

where *C*(*x*, *t*) is the concentration of a passive marker of particles. Equation (10) is solved in parallel to the Navier–Stokes Eq. (3) starting from the initial condition $C(x,0)=1$ at the beginning of diastole. The average concentration starts from a unitary value and decreases progressively every heartbeat when the marked particles are replaces by fresh blood entered from the atrium. In order to create a link with the existing literature in 4D Flow MRI, the flow transit is calculated in terms of direct and residual volume^48^. $V_{residual}$ is obtained with Eq. (10) from the evaluation as the following end systole

11 $$\begin{aligned} V_{residual}=\int _{ESV}CdV. \end{aligned}$$

$V_{direct}$ instead is calculated through the linear combination with the sub-volumes of Eq. (9) as widely discussed in a previous work^10^.

### Polynomial chaos expansion

Let $f(\cdot )$ denote a HFM predicting a set of QoIs, $y_k$, as a function of a vector ${\mathbf {p}} = \{p_1,\ldots ,p_M\}$ including key governing parameters subject to variability. If the variance of $y_k$ is finite, the PCE approximation, namely ${\hat{y}}_k$, holds and reads as follows^24,25^:

12 $$\begin{aligned} {\hat{y}}_k = \sum _{ \mathbf {a} \in {\mathbb {N}}^M} s_\mathbf {a} \Psi _\mathbf {a} (\mathbf {\xi }). \end{aligned}$$

In (12), multi-indices $\mathbf {a} = \lbrace a_1,\ldots , a_M \rbrace \in {\mathbb {N}}^M$ are associated with multivariate polynomials $\Psi _{\mathbf {a}}$ of degree $\left| \mathbf {a} \right| = \sum _{i=1}^{M} a_i$, which constitute an orthonormal basis with respect to the joint PDF of $\mathbf \xi$; $\xi _i$, with $i=1,\ldots ,M$, are independent standard random variables, associated with the governing parameters collected in ${\mathbf {p}}$ through an isoprobabilistic transform^42^; coefficients $s_\mathbf {a}$ are deterministic coordinates of the spectral decomposition^25^.

In practical applications, the PCE (12) is opportunely truncated as

13 $$\begin{aligned} {\hat{y}}_k = \sum _{i=0}^{P-1} s_i \Psi _i (\mathbf {\xi }), \qquad P = \frac{( M+q )!}{M!q!}, \end{aligned}$$

where *q* is the maximum degree of the expansion, i.e., $| \mathbf {a} | \le q$ for all $\mathbf {a} \in {\mathbb {N}}^M$. As a result of the approximation of (12) to polynomials of degree not exceeding *q*, the series reduces to a number of terms equal to *P* which depends on *q* and *M* as shown in (13).

Calibration of the PCE is performed using a non-intrusive approach based on regression, upon minimization of the variance of the residual $\varepsilon = |{\hat{y}}_k-y_k|$, with respect to the vector of unknown coefficients $s_i$^42^. The number of regression points is generally $N \ge P$. The optimum set of regression points in the standard normal space is provided by the PCM^43^, with the idea of employing the combinations of the roots of the next higher-order polynomial, i.e. $q+1$, as the points at which the minimization is solved. As such, the dataset $y_k$ is generated by computing the HFM at the regression points, while ${\hat{y}}_k$ is the approximation provided by the PCE at the same points. The accuracy of the PCE approximation is generally assessed by comparison with the HFM against a set of points in the parameter space not used during calibration. If the accuracy provided is not adequate, one can evaluate to improve the approximation by increasing the order of the expansion, *q*, which in turn increases the number of terms *P* in (13).

Moments of the QoI are estimated as follows^42^:

14 $$\begin{aligned} M\hat{y_k} = s_0, \qquad V\hat{y_k} = \sum _{i=1}^{P-1} s_i^2 \left\langle \Psi _i^2 (\mathbf {p}) \right\rangle . \end{aligned}$$

Similarly, computation of global sensitivity metrics, such as the Sobol’ indices^44^, consists in analytical post-processing of the PCE coefficients^26^, thus drastically reducing the associated computational cost. The use of the surrogate (12) sensibly accelerates MCS for risk analysis purposes, thus allowing for the estimate of the probabilistic behavior of the QoI^27^.

## Data Availability

All data generated or analysed during this study are included in this published article.
